# Creep reference data of single-crystal Ni-based superalloy CMSX-6

**DOI:** 10.1016/j.dib.2025.112436

**Published:** 2026-01-07

**Authors:** Luis Ávila Calderón, Sina Schriever, Ying Han, Jürgen Olbricht, Pedro Dolabella Portella, Birgit Skrotzki

**Affiliations:** a Federal Institute for Materials Research and Testing (BAM), Unter den Eichen 87, 12205 Berlin, Germany; b Fraunhofer-Institut für Werkstoffmechanik IWM, Wöhlerstraße 11, 79108 Freiburg, Germany

**Keywords:** Ni-based superalloy, Single-crystal, High temperature, Creep

## Abstract

The article presents creep data for the single-crystal, [001]-oriented nickel-based superalloy CMSX-6, tested at a temperature of 980 °C under initial stresses ranging from 140 MPa to 230 MPa. The constant-load creep experiments were performed in accordance with DIN EN ISO 204:2019–4 standard within an ISO 17025 accredited laboratory. A total of 12 datasets are included, each of which includes the percentage creep extension as a function of time. The data series and associated metadata were systematically documented using a data schema specifically developed for creep data of single-crystal Ni-based superalloys. This dataset serves multiple purposes: it can be used to compare with one's own creep test results on similar materials, to verify testing setups (e.g., by replicating tests on the same or comparable materials), to calibrate and validate creep models, and to support alloy development efforts.

Specifications Table

**Table:** 

Subject	Engineering & Materials science
Specific subject area	Mechanical behavior of single-crystal Ni-based superalloys under creep
Type of data	Table, Chart, Graph Raw, Analyzed, Processed
Data collection	Data were measured using a constant load creep testing machine with a 20 kN capacity (Mohr & Federhaff AG, Mannheim, Germany), equipped with a 2-zone furnace (each PID controlled) and a single-sided extension measurement (extensometer from MTS Systems, model 632.53F-31; measuring length: 25 mm).
Data source location	Federal Institute for Materials Research and Testing (BAM), Berlin, Germany
Data accessibility	Repository name: Zenodo Data identification number: 10.5281/zenodo.13937986 Direct URL to data: https://zenodo.org/records/14750432
Related research article	None

## 1 Value of the Data

• The characterized Ni-based superalloy CMSX-6 is of high technical relevance for high-temperature applications.
• The dataset contains 12 creep tests at 980 °C and initial stresses between 140–230 MPa, with three tests per stress level, and exhibiting high repeatability at identical test parameters.
• Researchers, engineers, and materials scientists may use this dataset as a benchmark to assess creep test results on similar materials and to verify testing setups, for example, by replicating creep tests on the same or comparable alloys.
• The dataset also enables the calibration and validation of creep models, supports alloy development efforts, and aids comparative material selection processes.
• To facilitate reuse, the data are provided in machine-readable formats and documented through a data-schema-based, structured, and comprehensive approach.

## 2 Background

CMSX-6 belongs to the first generation of single-crystal nickel-based superalloys, which were developed in the 1980s as a low-density alloy for use in turbine blades [1,2]. Unlike polycrystalline or directionally solidified materials, single-crystal alloys show higher creep resistance. This is, on the one hand, due to the lack of grain boundaries, which means that grain boundary sliding as a creep mechanism is not relevant. On the other hand, by eliminating grain boundary strengthening elements, the composition of the alloy can be adjusted to achieve a higher γ'-phase fraction (> 60 %), which results in strengthening. CMSX-6 has already been well characterized in the past regarding its microstructure and physical and mechanical properties [[3], [4], [5], [6], [7], [8]].

As part of the use case “Development and application of a framework for distribution of reference datasets” [9] in the NFDI-MatWerk project [10], published creep data of Ni-based superalloys and other alloys were examined in detail regarding the quality of their documentation. It was found that, despite the technical relevance of creep data, the documentation of published data is often insufficient. This frequently prevents the simple reuse of this data. As part of the use case, a reference data schema for single-crystal Ni-based alloys was therefore developed [11], which can also be used for research data and for other metallic materials [12]. For a distinction between *reference* and *research* data, see [13]. To illustrate how research and reference data in the field of materials science and engineering can be processed using the proposed framework, the concepts developed were prototypically implemented based on a specific example of the creep test of CMSX-6 [13], and a reference data set was published [14]. This article provides a detailed description of the experimental generation and documentation of the reference data.

## 3 Data Description

The dataset presented here qualifies as *reference* data of materials (see [15] for the definition and [13] for a discussion of the data management strategy). To structure and document the data, a recently developed data schema for documenting reference data from creep tests on single-crystal Ni-based superalloys, and which can be found in [11], was employed. The name of the data schema [11] follows the format “Data-Schema_Creep_vX.Y”, where X.Y denotes the version number. The data schema differentiates between metadata, primary, and secondary data (see column labeled as “Category I” in the data schema). More specifically, it includes comprehensive metadata covering the test procedure, the material tested, the test piece, the measuring and test equipment used, and the data processing procedures, along with test results, which represent primary and secondary data (see column labeled as “Category II” in the data schema). Further, more specific classifications are specified in the columns labeled as “Category III” and “Category IV”. The column labeled as “Entry” outlines the requested individual pieces of information. In the columns that follow to the right, the symbol, unit, and data type are specified. The column labeled as “Requirement” contains the requirement profile, as specified in [11,13]. An overview of the main categories of the data schema is presented in Table 1.

**Table 1:** Main categories of the data schema that was employed to structure and document the data [11]. This categorization corresponds to v1.1 of the data schema. More details about the categorization can be found in [11,13].

Category I	Category II
Metadata	Test info
	Material related
	Measuring and test equipment
	Data processing procedures
Primary data	Test result
Secondary data	Test result

**Fig. 1:**
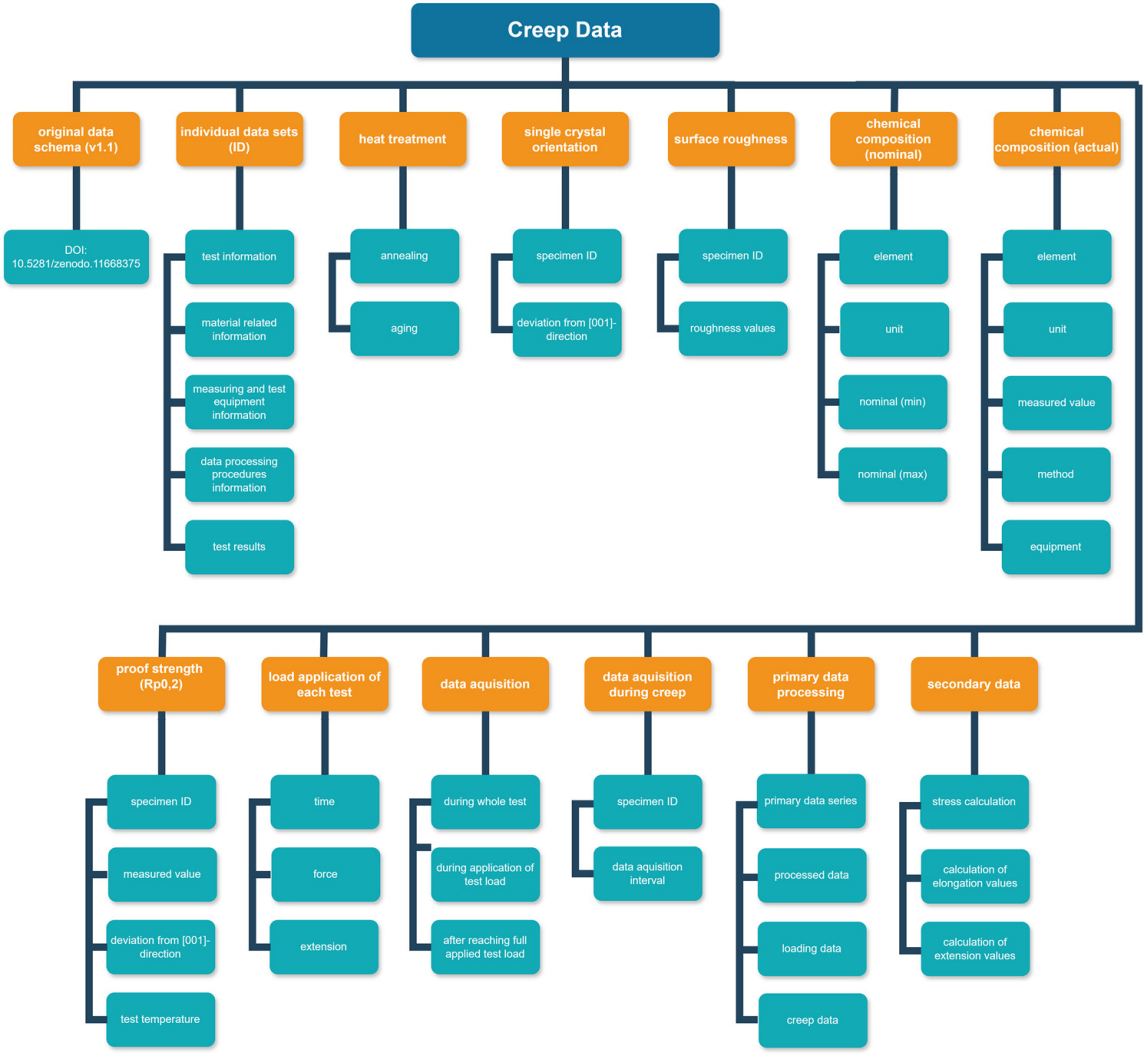
Overview of the data structure within the XLSX file provided in the dataset publication [14]. This structure corresponds to v1.0.

The dataset provided comprises several files: a) XLSX file with metadata, primary and secondary data, b) *.lis files with metadata and test results, and c) PDF files with information on test piece geometry and sampling, and microstructure. The data structure as delivered in XLSX file format in [14], which is based on the used data schema [11], is schematically represented in Fig. 1. The name of the accompanying XLSX file [14] follows the format “Creep-reference-data-CMSX-6_vX.Y.xlsx”, where X.Y denotes the version number. The data structure presented in Fig. 1 corresponds to v1.1. The XLSX file (version v1.0) incorporates the data schema [11] within the worksheet “Data-Schema-Creep”, and worksheets named according to the respective test IDs. These test-specific worksheets populate the column that follows the “Requirement” column with responses corresponding to the individual schema entries. In certain cases, these responses reference to additional worksheets within the same XLSX-file, which contain the associated metadata. Information that is neither contained in the worksheets named according to the respective test IDs nor in the additional worksheets within the XLSX file is contained in additional PDF files that are also attached to the data publication [14]. These additional files provide detailed information on the as-tested material as well as documentation of the test pieces, including provenance details from the material to be tested, and the geometry and dimensions of the test piece.

**Fig. 2:**
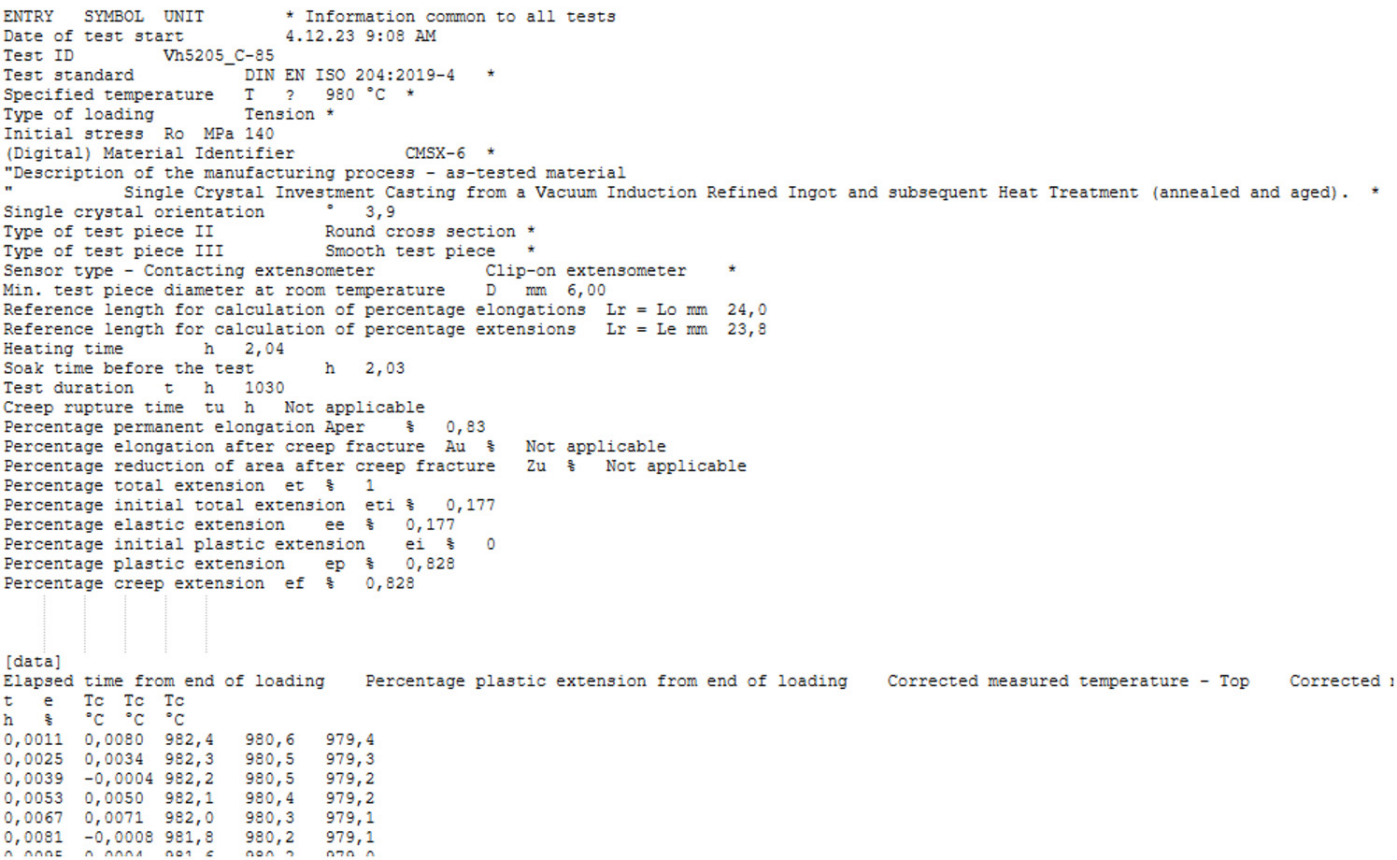
Excerpt of a *.lis file showing the header (top) and the processed data series (bottom). Note that (according to national convention) a comma is used as the decimal separator.

In addition to the XLSX and PDF files, in [14], individual *.lis files in ASCII format are provided for each test. These files include a header section containing selected metadata and test results, followed by time-resolved data series comprising time, creep percentage extension, and temperature. The data series provided in the *.lis files constitutes processed data. Details regarding the data processing are delivered in the next section of this manuscript. An excerpt of a *.lis file is exemplarily shown in Fig. 2. Apart from the creep deformation processed data series in the corresponding *.lis file for each test piece, processed data series from the loading phase are provided in a worksheet within the XLSX file. Data series from the heating-up and cooling-down phases are not provided, but the corresponding heating and soak times are reported. Further details regarding the primary and processed data series are provided in the XLSX file (see also Fig. 1).

Overall, the decimal point setting of test results adheres to DIN EN ISO 204:2019–04 [16] and reflects the resolution capabilities of the measuring devices used. The measurement uncertainties associated with the test temperature, T, the initial stress, $R_{o}$, and the percentage extension (i.e., strain), e, were quantified in accordance with the approach specified in CWA 15261–3:2005 [17], and are summarized in Table 2.

**Table 2:** Combined standard uncertainties (at 68 % confidence level) for the test temperature, T, initial stress, $R_{o}$, and percentage extension, e.

± $u_{c}$ % (T) [ °C]	± $u_{c}$ % ($R_{o}$) [MPa]	± u % (*e*) [%]
%	%	%
max. 0.3	max. 0.67	1.0

**Fig. 3:**
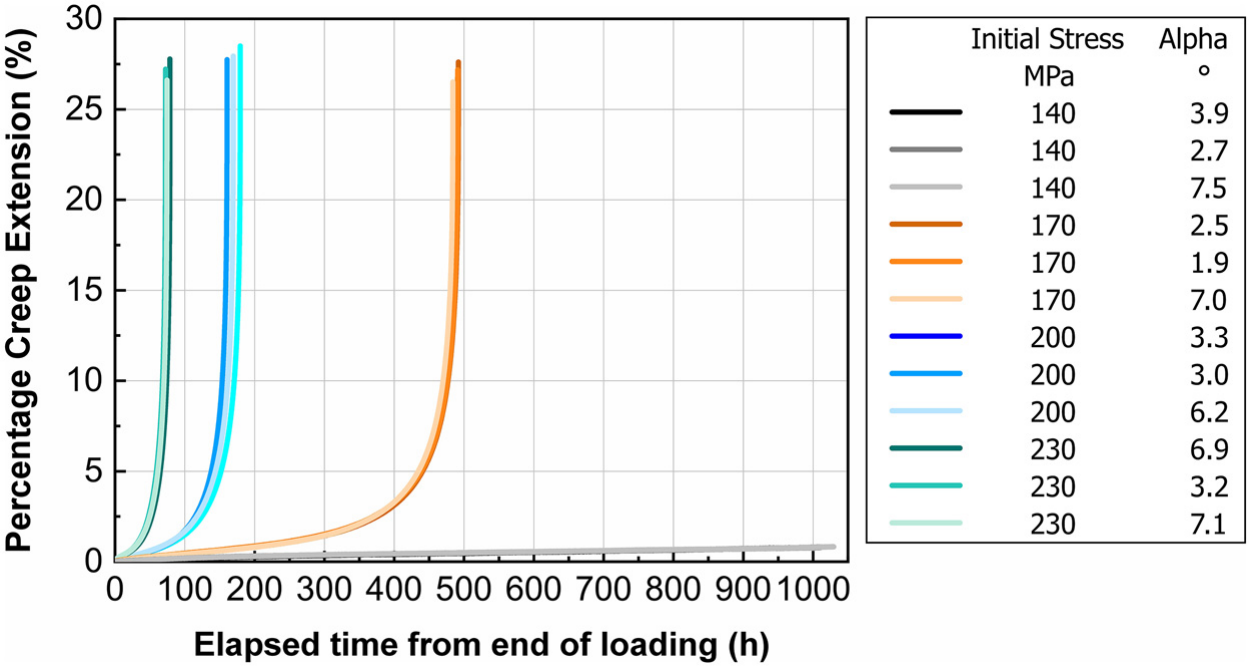
Creep extension (%) vs. time (h). T = 980 °C and different initial stresses. The legend also provides the deviation angle a from the [001] direction for each test piece. Three tests were performed at each stress level. Tests at 140 MPa initial stress were interrupted after around 1000 h; all other tests were terminated by fracture of the test piece.

Fig. 3 shows the percentage creep extension (i.e., creep strain), $e_{f}$, plotted vs. time, t, for the investigated temperature T = 980 °C and the four initial stresses. Three tests were performed at each stress level. Table 3 presents creep rupture times, $t_{u}$, and percentage elongation after creep fracture values, $A_{u}$, with their respective standard deviations. The results show a low scatter at identical test parameters, which indicates the robustness of the measurement setup and the homogeneity of the tested material. This low scatter is evident from the close grouping of the curves in Fig. 3 and is quantitatively supported, if applicable, by the standard deviations in Table 3.

**Table 3:** Creep rupture times, $t_{u}$, and percentage elongation after creep fracture values, $A_{u}$, with the respective standard deviations (SD) for all tested initial stresses, $R_{o}$, and orientations (Alpha angle).

$R_{o}$	Alpha	$t_{u}$	SD	$A_{u}$	SD
MPa	°	h	h	%	h
140	3.9	N/A		N/A	
140	2.7	N/A		N/A	
140	7.5	N/A		N/A	
170	2.5	492	4.4	27	0.6
170	1.9	491		27	
170	7.0	484		26	
200	3.3	170	9.5	28	0.6
200	3.0	180		28	
200	6.2	161		27	
230	6.9	78.7	3.2	28	1.0
230	3.2	72.4		27	
230	7.1	74.5		26	

## 4 Experimental Design, Materials and Methods

### 4.1 Material

**Fig. 4:**
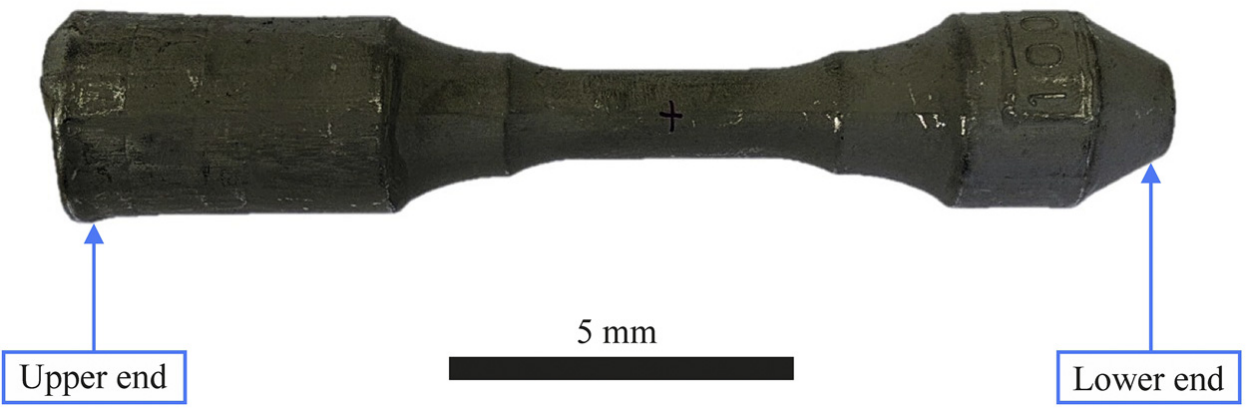
Example of a cast material after heat treatment.

The investigated specimens were produced by PCC Airfoils, Inc. (Minerva, OH) as investment castings from a vacuum induction refined ingot with a maximum diameter of approx. 30 mm and a height of approx. 150 mm. The blanks were cast in [001] orientation by directional solidification (Bridgman method) and subsequently heat-treated (solution annealed and aged); details of the multi-stage heat treatment process are given in [14]. The actual composition is given in Table 4 (for complete results, see [14]). Fig. 4 shows a cast blank in the heat-treated state. The absence of high-angle grain boundaries was verified by visual inspection of the investment cast's surface after electrolytic etching. The orientation was determined at the lower end (right side in Fig. 4) of each blank by the Laue method. The deviations of the [001] direction from the symmetry axis were documented in [14].

**Table 4:** Actual chemical composition (main elements) of CMSX-6.

Element	Cr	Co	Al	Ti	Mo	Ta	W	C	Hf	Other elements	Ni
wt. %	9.8	5	4.82	4.69	3	2	< 0.1	20 ppm	0.05	< 0.23	Balance

**Fig. 5:**
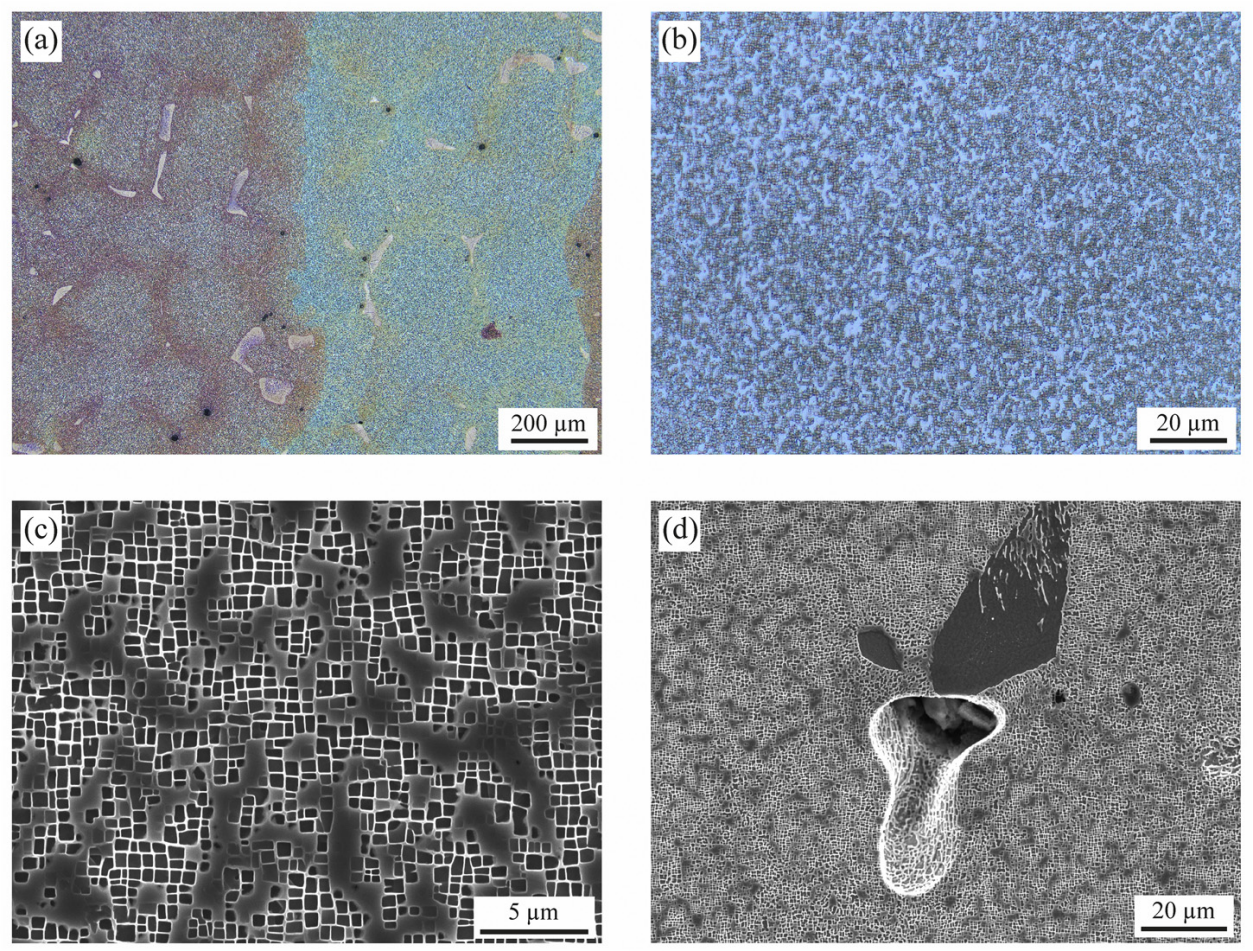
Microstructure in cross-section (i.e., perpendicular to the direction of loading) before creep testing. (a) Overview showing different dendrites with inter-dendritic and dendritic regions (optical microscopy). Between the reddish and bluish areas, a low-angle grain boundary runs from top to bottom, which is tolerated in single crystals. (b, c) Microstructure inside the dendrites in (b) low magnification (optical microscopy), and (c) higher magnification (SEM, SE-mode). (d) Interdendritic region at higher magnification. (SEM, SE-mode). Sample preparation: Grinding (SiC paper, grit sizes 180 / 320 / 600 / 1200); polishing (diamond paste, grit sizes 6 µm / 1 µm and Mastermet II, SiO, 0.2 µm); etching (concentrate consisting of: 300 ml distilled water, 300 ml hydrochloric acid, 6 g molybdic acid; etching solution: 60 ml concentrate, 50 ml distilled water, 30 ml nitric acid).

Fig. 5 shows the microstructure of the specimens in the heat-treated state before creep testing. The metallographic specimens were prepared from a longitudinal section by conventional preparation methods. An overview with low magnification (Fig. 5a) shows a low-angle grain boundary from top to bottom. These subgrains are originated by small fluctuations in the temperature gradient during the directional solidification of the blank; the current specifications for so called single crystal components do not object the presence of subgrains. A great number of dendrites can be clearly identified by the secondary dendrites in [100] and [010] orientation. The microstructure inside the dendrites is homogeneous (Fig. 5b) with cuboidal precipitates of the γ’ phase well aligned in [001] directions (Fig. 5c). The dark grey patches are plates of the γ phase lying on the (001) plane. The interdendritic regions (Fig. 5d) solidify later than the surrounding dendrites; the composition of the remaining liquid phase shifts to a eutectic point, the final solidification leads to colonies of γ/γ’ eutectic. Shrinkage voids are also present in these regions; due to the growth of the primary dendrites and the intricate, slim channels between the secondary dendrites, the liquid phase ahead of the solidification front cannot feed all the islands of liquid phase in the interdendritic regions.

The 0.2 % proof stress, $R_{p0.2}$, determined in 5 tensile tests at creep test temperature (980 °C) varied between 390 MPa and 416 MPa. The creep specimens were extracted from the cast blanks such that the cast blank axis, the creep test piece axis, and the load application axis are mutually aligned along a common centerline (cf. Fig. 4). The center of gravity of the test piece was located on the longitudinal axis in the center area at the smallest diameter.

### 4.2 Creep testing

Creep tests were conducted in accordance with DIN EN ISO 204:2019-04 [16] under constant load conditions. The tests were terminated either by rupture of the test piece or once the minimum creep rate was reached. A modified 20 kN creep testing machine (Mohr & Federhaff AG, Mannheim, Germany) was used. The machine met calibration class 1 requirements for applied force as specified in DIN EN ISO 7500–2:2007-04 [18], and was equipped with a 2-zone furnace (ATS Applied Test Systems, Inc., USA, series 3330). The test load, corresponding to the full initial stress level, was applied smoothly without shock. Before heating, a preload of 0.2 kN was applied to ensure that the extensometer remained securely in place during the initial heating phase.

**Fig. 6:**
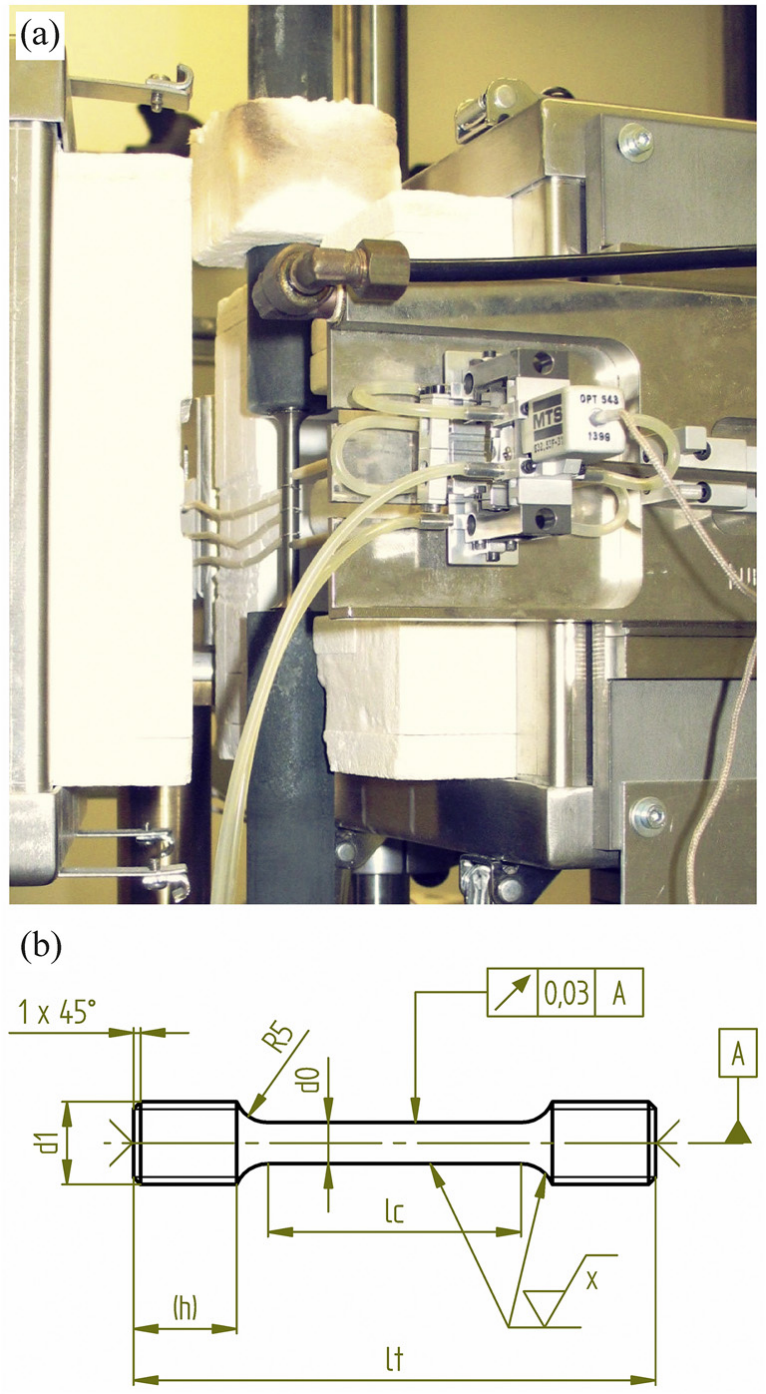
a) Creep test setup, b) technical drawing of the used creep test geometry.

Temperature data were recorded using calibrated SCXI-1102 acquisition units from National Instruments (National Instruments Kft., Debrecen, Hungary). Temperature control was managed via temperature signals from the furnace. To monitor the specimen temperature, three calibrated type S thermocouples were tied along the gauge length. Extension measurements were carried out using an MTS water-cooled, high-temperature, single-sided extensometer (Model 632.53F-31, class 1, nominal gauge length: 25 mm) from MTS Systems. An exemplary image of the test setup is shown in Fig. 6a.

The tests were conducted at a temperature of 980 °C, with initial stress levels set at 140 MPa, 170 MPa, 200 MPa, and 230 MPa. For each stress level, three individual tests were performed. The primary dataset consists of time (in seconds), displacement (in millimeters), and temperature (in degrees Celsius) readings recorded by each sensor.

Test specimens with a smooth, round cross-section were used. The gauge length was ground to a surface roughness of Rz ≤ 2.1. Each specimen featured M16 threaded ends, a total length (lt) of 76 mm, a gauge diameter (d0) of 6 mm, and a reduced parallel section length (lc) of 36 mm. The test piece geometry is shown in Fig. 6b.

### 4.3 Data processing

The creep deformation phase (data series provided in the *.lis files) begins immediately after the loading phase concludes. Time series data, originally recorded in seconds since the beginning of the test (heating phase), were converted to hours. The reference time point, t = 0 (corresponding to point b in Fig. 1 of [16]), marks the moment when the full initial stress was applied to the test piece. The original time value, which includes the heating-up phase, was reset to zero at that point. The temperature readings (in °C) reflect corrected values, which were adjusted based on deviations determined during the calibration processes of both temperature sensors and data acquisition unit. Extension values (mm) were converted to percentage extension values (%) using the appropriate reference length, which was determined at room temperature after mounting the extensometer under preload conditions, as specified in the test standard [16].Similar to the procedure followed for the creep deformation phase, in the processed data series that belong to the loading phase, which are provided in the XLSX file, the time series values (s) were reset to zero (*t* = 0) for better representation of the data.

The extension values provided both in the data schema and in the header of the *.lis files, were calculated based on Eqs. (1), (2), and (3) [16] (see also Fig. 1 in [16]). In accordance with the test standard, a distinction was made between the parameters obtained during the loading phase – namely, elastic extension ($e_{e}$), initial total extension ($e_{ti}$), and initial plastic extension ($e_{i}$) – and those considering the creep deformation phase, including total extension ($e_{t}$), plastic extension ($e_{p}$), and creep extension ($e_{f}$). To determine the extension values generated during the loading phase ($e_{e},\;e_{ti}$, and $e_{i}$), firstly, a linear fit was applied to the stress-extension data within the elastic region. A correction due to the preload was included. Subsequently, both $e_{e}$ and $e_{ti}$ are calculated as differences in extension values (mm) divided by the reference length: $e_{e}$ uses the values at the end of the elastic response and the beginning of loading, whereas $e_{ti}$ uses the values at the end and beginning of loading. $e_{i}$ can be then determined according to Eq. (1). For the values that consider the creep deformation, first, $e_{t}$ is determined as the difference in extension values (mm) at the end of the test (prior to unloading) and at the beginning of loading, divided by the reference length. Subsequently, *e_p_* and *e_f_* can be determined with Eqs. (2) and (3), respectively.

(1) $$e_{i}=e_{ti}-e_{e}$$

(2) $$e_{p}=e_{t}-e_{e}$$

(3) $$e_{f}=e_{p}-e_{i}$$

## Limitations

None.

## Ethics Statement

The authors have read and followed the ethical requirements for publication in Data in Brief and confirm that the current work does not involve human subjects, animal experiments, or any data collected from social media platforms.

## CRediT Author Statement

**Luis Ávila Calderón:** Conceptualization, Validation, Formal analysis, Data curation, Visualization, Writing - Review & Editing; **Sina Schriever:** Investigation, Formal analysis, Data curation; **Ying Han:** Validation, Data curation; **Jürgen Olbricht:** Conceptualization, Writing - Review & Editing; **Pedro Dolabella Portella:** Data curation, Writing – Review & Editing; **Birgit Skrotzki:** Conceptualization, Supervision, Funding acquisition, Writing - Original Draft.

## Data Availability

ZenodoBAM Reference Data: Creep of Single-Crystal Ni-Based Superalloy CMSX-6 (Original data).
